# Persistent Pain and Purulent Discharge: A Case of Infected Bipolar Hemiarthroplasty

**DOI:** 10.7759/cureus.56375

**Published:** 2024-03-18

**Authors:** Kevin Kawde, Gajanan Pisulkar, Ankur Salwan, Adarsh Jayasoorya, Vivek H Jadawala, Aditya Chirayath

**Affiliations:** 1 Orthopaedics, Jawaharlal Nehru Medical College, Datta Meghe Institute of Higher Education and Research, Wardha, IND

**Keywords:** surgical intervention, purulent discharge, persistent pain, total hip arthroplasty, bipolar hemiarthroplasty, prosthetic joint infection

## Abstract

Prosthetic joint infection (PJI) remains a significant complication following joint arthroplasty, necessitating prompt recognition and intervention to optimize patient outcomes. This case report describes a 65-year-old male who presented with persistent pain, swelling, and purulent discharge from the right hip, three years post-bipolar hemiarthroplasty following a road traffic accident. Clinical examination revealed signs suggestive of PJI, prompting surgical intervention with total hip arthroplasty. Postoperatively, the patient experienced resolution of symptoms and satisfactory recovery. This case underscores the challenges associated with infected joint arthroplasty and highlights the importance of a multidisciplinary approach for effective management. Early diagnosis, appropriate surgical intervention, and comprehensive postoperative care are essential for minimizing morbidity associated with PJIs and optimizing patient outcomes.

## Introduction

Joint arthroplasty, particularly bipolar hemiarthroplasty, is a commonly performed surgical procedure for various hip pathologies, including fractures and degenerative joint diseases [1]. While these surgeries generally have favorable outcomes, complications such as infection can significantly impact patient morbidity and mortality rates. Infection following joint arthroplasty is a serious concern, with reported incidence rates ranging from 1% to 2% [1,2]. It can manifest as acute or chronic, with symptoms including pain, swelling, erythema, and purulent discharge from the surgical site [3]. Persistent infection can lead to implant loosening, osteomyelitis, and systemic complications such as septicemia.

Prompt recognition and management of infected joint arthroplasty are crucial for preventing adverse outcomes. Clinical suspicion, supported by laboratory investigations, including erythrocyte sedimentation rate and C-reactive protein levels, along with imaging modalities such as X-rays and magnetic resonance imaging (MRI), aid in diagnosis [4]. Treatment of infected joint arthroplasty typically involves a multidisciplinary approach, including surgical intervention and antibiotic therapy. Surgical options may include debridement and implant retention, one-stage or two-stage revision arthroplasty, or, in severe cases, resection arthroplasty [5]. Total hip arthroplasty (THA) may be indicated in cases of persistent infection to eradicate the source and restore joint function.

## Case presentation

A 65-year-old male presented to the orthopedic clinic with complaints of persistent pain and swelling over his right hip, which he had been experiencing for the past three years. The patient reported a history of a road traffic accident three years prior, for which he underwent a surgical procedure involving bipolar hemiarthroplasty at a government hospital in his native place. Despite initial relief following the surgery, the patient started experiencing pain, swelling, and purulent discharge from the surgical site approximately one year postoperatively. The symptoms progressively worsened, leading to difficulty in weight-bearing and ambulation.

The patient’s pain was described as continuous, moderate in intensity, and exacerbated by movement. It was associated with a mild, yellowish, purulent discharge from the surgical site. Pain relief was achieved with rest and analgesic medications. The patient reported limitations in performing daily activities due to the severity of pain and swelling. Additionally, he experienced two episodes of fever per month over the past year. There was no history of vomiting, head injury, loss of consciousness, or any significant medical comorbidities such as diabetes mellitus, hypertension, or tuberculosis.

**Figure 1:**
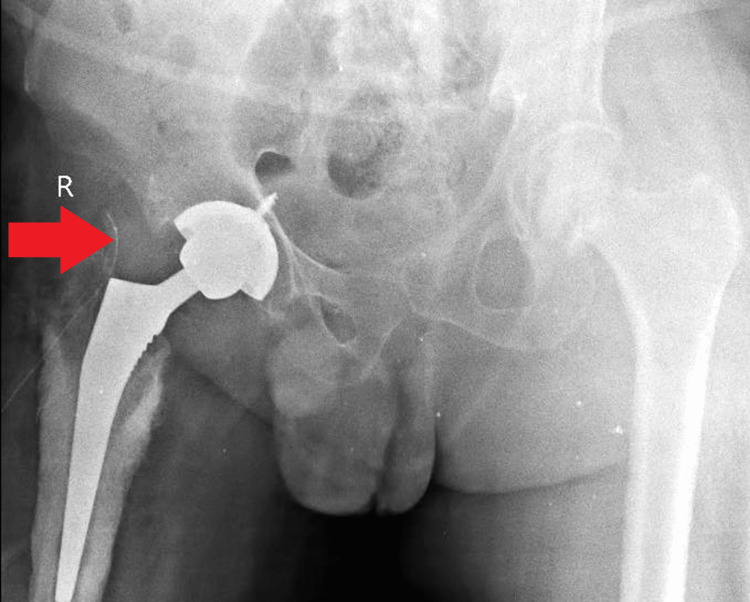
Image showing right-sided bipolar hemiarthroplasty.

On physical examination, inspection revealed swelling over the right hip and thigh region with scars from the previous surgery, approximately 8 × 1 cm in size, present over the lateral aspect of the thigh. No discharging sinus or dilated veins were observed. Palpation revealed localized tenderness and increased local temperature over the right hip compared to the contralateral side. Range of motion at the hip joint was limited due to pain, and the patient preferred to keep the hip in extension, knee in extension, patella facing upwards, and ankle in plantar flexion. Sensory function and distal circulation were intact, and active ankle and toe movements were preserved. Given the history of persistent infection and clinical findings suggestive of an infected bipolar hemiarthroplasty, the decision was made to proceed with THA on the affected side (Figure 1).

**Figure 2:**
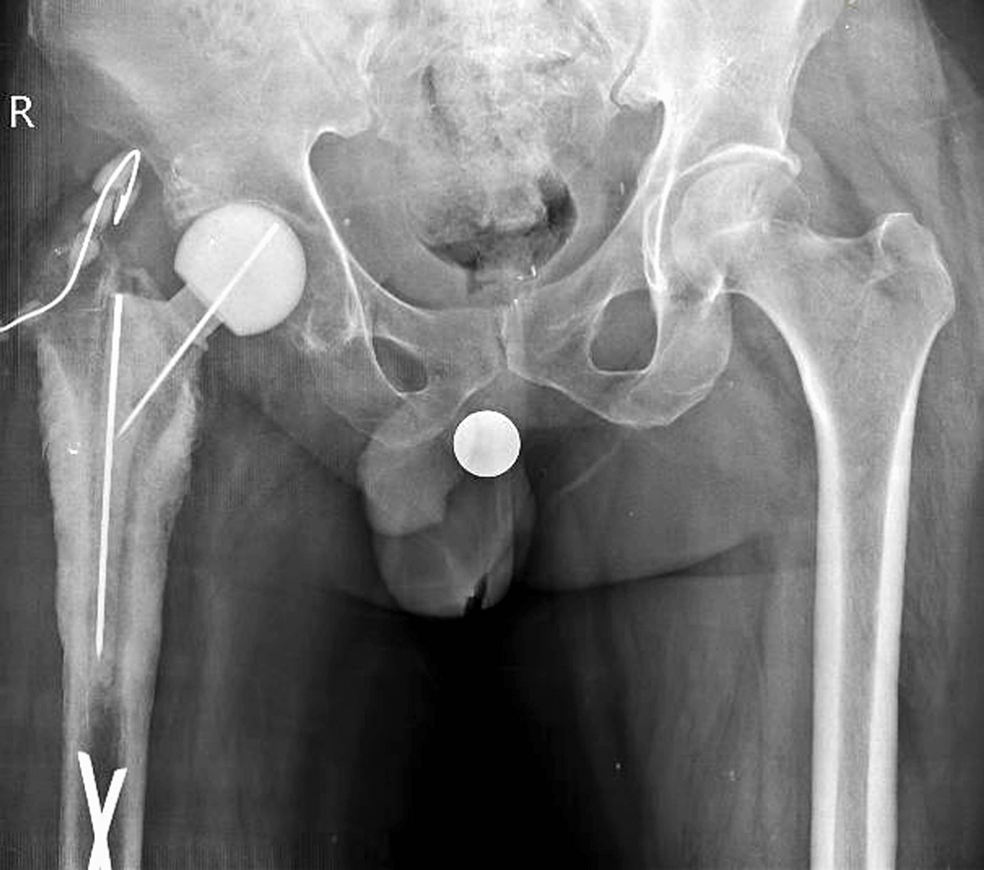
Postoperative image showing components of appropriate size selected and securely fixed in place.

The surgical procedure was performed under spinal and epidural anesthesia with meticulous attention to the aseptic technique. An approximately 8 cm curved incision was made over the previous surgical scar, followed by dissection through the superficial and deep fascia. The acetabular cavity was prepared, and the femoral canal was accessed and prepared accordingly. Components of appropriate size were selected and securely fixed in place. The surgical site was irrigated thoroughly, and closure was performed in layers (Figure 2).

Postoperatively, the patient’s course was uneventful, with a resolution of pain and swelling over the right hip. Physical therapy and rehabilitation were initiated to facilitate recovery and regain functionality. Follow-up evaluations indicated satisfactory healing and restoration of hip function. The patient was discharged with appropriate instructions for wound care, activity modification, and follow-up appointments to monitor for any signs of infection or complications.

## Discussion

Infection following joint arthroplasty is a significant complication that can lead to persistent pain, functional impairment, and implant failure if not promptly recognized and managed [4]. The presented case highlights the challenges associated with infected bipolar hemiarthroplasty and underscores the importance of timely intervention to optimize patient outcomes. Early prosthetic joint infection (PJI) diagnosis remains a clinical challenge due to nonspecific symptoms and imaging findings [6]. In this case, the patient’s persistent pain, swelling, and purulent discharge raised suspicion for PJI, prompting further evaluation. Clinical examination, supported by laboratory tests including serum inflammatory markers and joint aspiration for culture and analysis, is crucial in confirming the diagnosis [7]. Imaging modalities such as plain radiographs, ultrasound, and MRI may aid in identifying signs of infection, including peri-prosthetic fluid collections and osteolysis [8].

Once PJI is suspected or confirmed, prompt intervention is essential to eradicate infection and prevent further complications. Surgical management, including debridement and implant retention, exchange arthroplasty, or two-stage revision, aims to remove infected tissues, restore joint function, and prevent recurrence [9]. In this case, the decision to proceed with THA was based on the severity of the infection and the patient’s clinical presentation. THA offers the advantage of complete removal of infected components, thorough debridement of necrotic tissues, and placement of new implants, thereby reducing the risk of persistent infection [10]. Following surgical intervention, postoperative care and rehabilitation are essential for promoting recovery and optimizing functional outcomes. Physical therapy aims to improve joint mobility, muscle strength, and gait mechanics, facilitating early ambulation and return to activities of daily living [11]. Close monitoring for signs of recurrent infection, such as persistent pain, swelling, or systemic symptoms, is necessary during the postoperative period [12]. Long-term follow-up is recommended to assess the durability of the implants and monitor for late complications, including aseptic loosening and implant wear [13].

## Conclusions

The case of infected bipolar hemiarthroplasty presented here underscores the challenges associated with PJIs and highlights the importance of prompt recognition and intervention. Through a multidisciplinary approach involving clinical evaluation, laboratory investigations, and surgical management, the patient achieved resolution of symptoms and satisfactory recovery following THA. Long-term follow-up and vigilant monitoring are essential to assess the durability of the implants and detect any late complications. This case emphasizes the significance of early diagnosis, appropriate surgical intervention, and comprehensive postoperative care in optimizing outcomes and minimizing morbidity associated with PJIs. Continuing research efforts and advancements in diagnostic modalities and treatment strategies are necessary to further improve the management of infected joint arthroplasty and enhance patient outcomes.
